# Metropolitan Hospital

**Published:** 1905-07-15

**Authors:** 


### METROPOLITAN HOSPITAL.

THE Hon. Claude G. Hay, M.P., presided at the festival dinner of the Metropolitan Hospital, which was held at the Whitehall Rooms on Tuesday evening (July 11th), and was supported by Lord Howard de Walden (the Chairman of the hospital), Sir J. Heron Maxwell, Bart., Mr. Leopold de Rothschild (Treasurer), Sir Edmund Hay Currie, etc.

The Chairman, in proposing "Prosperity to the Hospital," referring to the conditions under which Londoners lived, said that personal service in London was almost an impossibility. One did not know one's neighbour. Wapping and Wandsworth, Mile End and Marylebone, were almost as far apart in their daily life as Cornwall and the uttermost parts of Northern Scotland. Therefore it was in institutions such as the Metropolitan Hospital that they found that possibility which good men sought of rendering service to their fellow-men. In appealing for the Metropolitan Hospital they were often met by the remark, "Oh, but this is a City hospital; let the City find the money for it." The reply to that was that the whole City either came from the West End or went to the West End, and therefore the West End could not divorce itself from its responsibilities to an institution which had its origin within the bounds of the City.

Lord Howard de Walden, in reply, said that the hospital had many needs, the chiefest of which was an absolutely able-bodied philanthropist.

Sir E. Hay Currie, in proposing "The Committee of Management," said he thought that toast was if anything even more important than the previous one, for the prosperity of the hospital depended more largely upon its committee than on anything else. The Metropolitan Hospital had everything in that way that was required. It had an excellent Chairman; it had a committee composed of men of various walks of life, who gave to it their time and power in dealing with such questions as might from time to time arise; and lastly it had, he believed, one of the best Secretaries in London. (Applause.) He supposed there never was a time in the history of the hospitals of London when it was more necessary for those who cared for the hospitals to put their shoulders to the wheel in their behalf. Last year the difference between the income and the expenditure of the hospitals of London, excluding Bartholomew's, was something like a quarter of a million; that was to say, that, including legacies, the income of the hospitals was no less than £250,000 less than the expenditure. The question therefore was, how were they to raise that gigantic sum of money? One way was by the exercise of rigid economy, by doing everything to bring down the expenditure without in so doing impairing their efficiency. He knew from his position as Hon. Secretary of the Hospital Sunday Fund that the hospitals of London were doing everything they could to reduce their expenditure. It was absolutely necessary that they should try and save a very considerable amount of money in order to get their income on an equality with their expenditure.

Mr. C. J. Thomas, Chairman of the Committee of Management, replied.

During the evening a collection of £3,825 was announced as the result of the dinner.

